# InterProScan 5: genome-scale protein function classification

**DOI:** 10.1093/bioinformatics/btu031

**Published:** 2014-01-29

**Authors:** Philip Jones, David Binns, Hsin-Yu Chang, Matthew Fraser, Weizhong Li, Craig McAnulla, Hamish McWilliam, John Maslen, Alex Mitchell, Gift Nuka, Sebastien Pesseat, Antony F. Quinn, Amaia Sangrador-Vegas, Maxim Scheremetjew, Siew-Yit Yong, Rodrigo Lopez, Sarah Hunter

**Affiliations:** ^1^European Molecular Biology Laboratory, European Bioinformatics Institute (EMBL-EBI), Wellcome Trust Genome Campus, Hinxton CB10 1SD and ^2^Wellcome Trust Sanger Institute, Wellcome Trust Genome Campus, Hinxton CB10 1SA, UK

## Abstract

**Motivation:** Robust large-scale sequence analysis is a major challenge in modern genomic science, where biologists are frequently trying to characterize many millions of sequences. Here, we describe a new Java-based architecture for the widely used protein function prediction software package InterProScan. Developments include improvements and additions to the outputs of the software and the complete reimplementation of the software framework, resulting in a flexible and stable system that is able to use both multiprocessor machines and/or conventional clusters to achieve scalable distributed data analysis. InterProScan is freely available for download from the EMBl-EBI FTP site and the open source code is hosted at Google Code.

**Availability and implementation:** InterProScan is distributed via FTP at ftp://ftp.ebi.ac.uk/pub/software/unix/iprscan/5/ and the source code is available from http://code.google.com/p/interproscan/.

**Contact:** http://www.ebi.ac.uk/support or interhelp@ebi.ac.uk or mitchell@ebi.ac.uk

## 1 INTRODUCTION

The InterProScan software (Quevillon *et al.*, 2005) is extensively used both by genome sequencing projects [Suen *et al.*, 2011; Shulaev *et al.*, 2011; Sato *et al.*, 2011] and the UniProt Knowledgebase (UniProtKB) (The UniProt Consortium, 2012) to obtain a ‘first-pass’ profile of protein sequences’ potential functions. It does this by combining together search applications that predict protein family membership and the presence of functional domains and sites, summarizing their outputs.

**Fig. 1.**
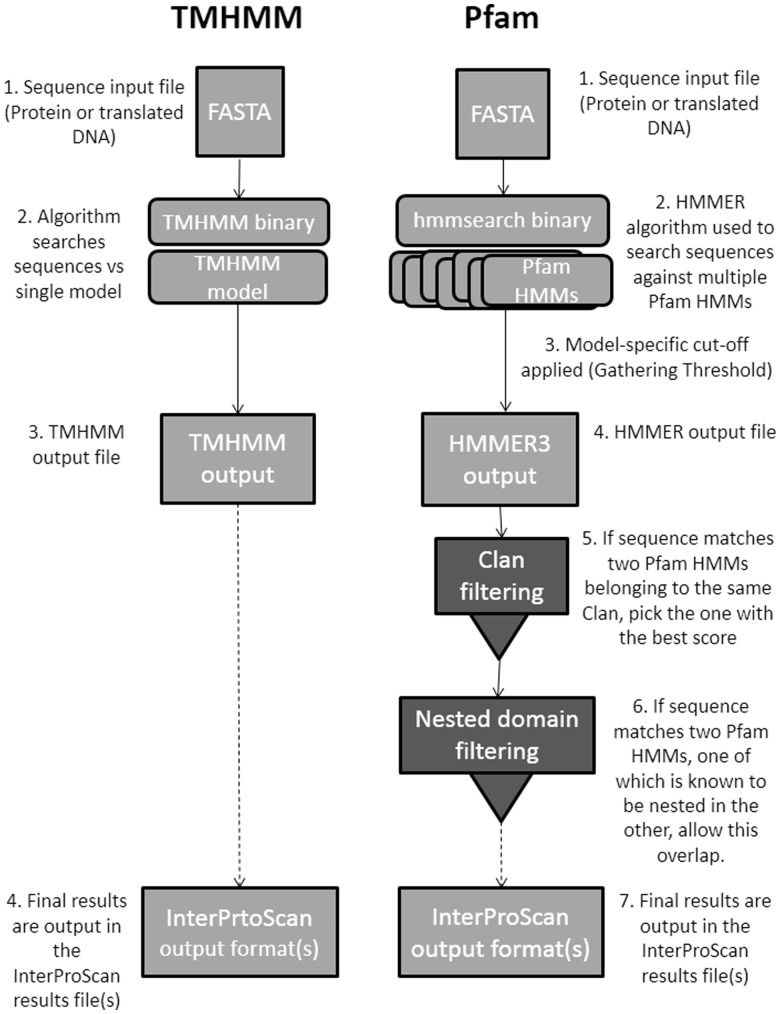
Comparison of the processing steps used by two different member database applications, TMHMM and Pfam

Before describing the architecture used by the new version of InterProScan, it is necessary to explain how these analysis applications work in a general sense, as it has influenced the overall design of the system. Search applications have two main modalities: The simplest [TMHMM (Krogh *et al.*, 2001), SignalP (Petersen *et al.*, 2011), Phobius (Käll *et al.*, 2004)] have single algorithms that are used to give a likelihood that a particular feature exists. Other applications [Pfam (Punta *et al.*, 2012), TIGRFAMs (Haft *et al.*, 2012), SMART (Letunic *et al.*, 2012), PIRSF (Wu *et al.*, 2004), Panther (Mi *et al.*, 2012), HAMAP (Pedruzzi *et al.*, 2012), Prosite (Sigrist *et al.*, 2012), ProDom (Bru *et al.*, 2005), PRINTS (Attwood *et al.*, 2012), CATH-Gene3D (Lees *et al.*, 2012) and SUPERFAMILY (De Lima Morais *et al.*, 2011)] are more complex, searching a sequence against multiple models, using a variety of algorithms [e.g. HMMER (Eddy, 2009), BLAST (Altschul *et al.*, 1990)] and post-processing the raw output of the search algorithm before a final result is produced (see Fig. 1). Frequently, the algorithms used are computationally expensive and some kind of parallelization is necessary for large numbers of sequences to be analyzed in a reasonable amount of time. Once the search results are obtained, the InterProScan in-memory database is queried to find corresponding InterPro (Hunter *et al.*, 2012) entries (i.e. the entries associated with a particular set of HMMs, etc) and additional database annotations, such as Gene Ontology (The Gene Ontology Consortium, 2000) terms, are associated with the results accordingly.

It is imperative that the framework built for managing the InterProScan analyses is highly configurable, so that the heterogeneous nature of the different applications can be represented appropriately, and the software can run in a multitude of computing environments. Previous versions of InterProScan (4.*x* and earlier) were difficult for users to install and configure, did not scale appropriately for the analysis of large numbers of sequences and the functionality of the software was difficult to extend. This reimplementation of InterProScan addresses the previous versions’ weaknesses and adds new features to the software.

## 2 SOFTWARE ARCHITECTURE

The design goals for InterProScan 5 are driven by a wider set of use cases than existed for InterProScan 4 and earlier versions of the software package. In common with earlier versions, InterProScan 5 has been designed to allow the efficient characterization of relatively small numbers of sequences in a single analysis, including the ability to parallelize search jobs to minimize wall-clock time (as opposed to CPU time).

New to InterProScan 5 is the ability to function on a massive scale, to allow the analysis and persistence of match data for millions of sequences under the control of a single ‘Master’ process with a high level of parallelization of the analysis steps on a cluster or supercomputer. This parallelization is done at three levels: sequence sets can be chunked into smaller sets for analysis (parallelization at the sequence level), individual analyses (e.g. Pfam, Prosite, etc) can run on separate threads on the same CPU, on different CPUs or on different machines (parallelization at the application level) and some application binaries, such as HMMER3, also take advantage of parallel computing (parallelization at the binary level). The large-scale mode makes use of a single relational database to store information about sequences (either nucleotide or protein sequences), predictive models and InterPro content, and the matches predicted by InterProScan. As InterProScan 5 makes use of the Hibernate object-relational mapping tool (http://www.hibernate.org/), it should be possible to port the back-end to run on most relational database management systems. By default, InterProScan 5 uses a pure in-memory database, which requires no configuration.

### 2.1 Overall system architecture

**Fig. 2.**
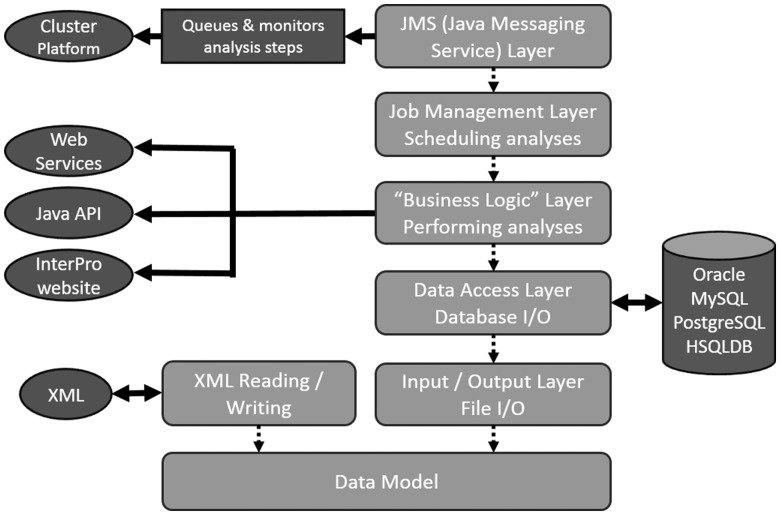
Overall system architecture of InterProScan 5

InterProScan 5 has a modular Java-based architecture, which builds on best-of-breed Java technologies. InterProScan is built on a rich Java data model that incorporates mappings to both a relational database schema (using Hibernate) and a new XML schema (using JAXB). Multiple layers are build on this core, each with different functionality (e.g. persistence of the data to a relational database, running of the analyses and farming out of jobs to computational resources, see Fig. 2).

### 2.2 Job management

Each analysis is defined as a ‘job’ in InterPro. A job may contain any number of steps that are defined with dependencies that allow merging and branching. Both jobs and steps are defined and wired together in an XML format using a mixture of common components (e.g. modules to write out FASTA files or run binaries) and analysis-specific components (e.g. modules to parse algorithm output formats or run analysis-specific post-processing).

**Fig. 3.**
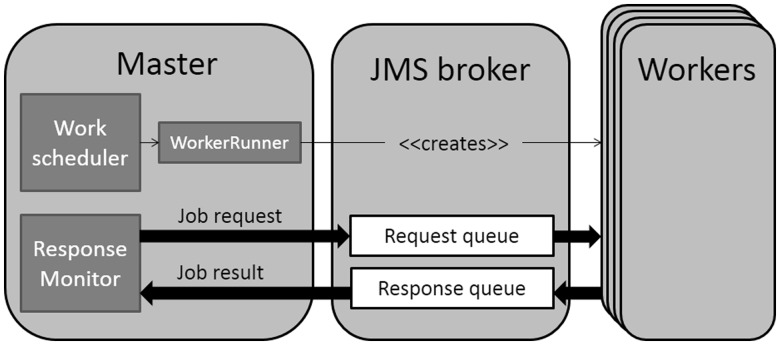
Use of JMS to manage allocation of jobs across a compute resource. This figure shows the primary tier of Master JVM-spawned workers. Jobs are added to a RequestQueue by the Master JVM, and any available worker JVMs will poll this queue to request work

InterProScan 5 makes use of Java Message Service (JMS) to manage communication between the components of the architecture, each of which may be run on separate physical or virtual machines (see Fig. 3). This communication system allows InterProScan 5 to be run on disparate environments, including a single machine, a local area network, a multi-core supercomputer or a managed cluster. The only requirement (at present) is that the machines share the same file system. JMS was selected after a careful review of multiple technologies for building and running distributed systems. Other evaluated technologies included MPI and Hadoop (http://hadoop.apache.org). MPI, although mature, did not have a well-supported official Java binding and Hadoop required a set up that was not compatible with all InterProScan use cases. JMS has proven to be robust, scalable and reliable for inter-process communications and is a stable industry standard (JMS version 1.1 was finalized in April, 2002). InterProScan 5 makes use of the open source Apache ActiveMQ JMS implementation (http://activemq.apache.org/).

A tiered hub/spoke architecture is used to allow InterProScan 5 to scale to potentially thousands of separate machines. At the center (hub) of the architecture is a single ‘Master’ process that dynamically spawns and manages a number of ‘Worker’ nodes. The Worker nodes consist of Java Virtual Machines (JVMs), which are able to perform multiple steps from different analyses during their lifetime. If additional nodes are required due to the load on the system, new ones will be spawned; once they have completed their tasks, they expire and free up resources. To avoid the problem of having too many open direct network connections between the Master and its Workers under heavy load, a Worker can itself spawn a new tier of Workers, if a set limit is reached; any number of tiers of Workers is allowed. Consequently, it is possible to run InterProScan 5 with tens of thousands of worker JVMs being controlled by a single Master JVM, allowing the analysis of entire genomes in just hours of wall-clock time.

To benchmark the software, we used InterProScan version 5.1–44.0 cluster mode to run all InterPro 44.0 member database and COILS analyses against the *Escherichia coli* DH10B proteome (3990 sequences) and *Arabidopsis thaliana* proteome (31 819 sequences). The wall-clock times were 32 and 520 min, respectively. The *E.coli* run used 12 workers, whereas the *Arabidopsis* run used 67 (each worker used four cores).

Another benefit of the JMS architecture is that it separates the functioning of the analysis software from the mechanics of the computer system that InterProScan 5 is running on. If, for example, a cluster system wishes to start a JVM on a new machine but fails to do so, InterProScan 5 is robust and will be unaffected by this failure. InterProScan has been developed to work with the cluster queuing management system IBM Platform LSF (http://www.ibm.com/systems/technicalcomputing/platformcomputing/products/lsf/), but its structure is generic enough to work with other systems, including Oracle Grid Engine (http://www.oracle.com/us/products/tools/oracle-grid-engine-075549.html).

### 2.3 New analysis algorithm and features

Besides the changes to the overall architecture and organization of the InterProScan software, a new analysis algorithm and new features have also been added. Phobius is a ‘combined transmembrane topology and signal peptide predictor’ (http://phobius.binf.ku.dk/). Its strengths include the ability to discriminate between the hydrophobic regions of a protein and signal peptides, which are highly similar. This application is freely available to academic users; however, it requires a license for commercial use. As a consequence, Phobius is not included in the downloadable version of InterProScan 5, but needs to be obtained separately from the Phobius Web site and configured for local use within InterProScan 5.

InterProScan summarizes the results obtained from running the separate search applications by checking the InterPro database to see which InterPro entries the matching signatures (i.e. the underlying models, such as HMMs, fingerprints and patterns) belong to and reporting these in the output. Signatures are only integrated into InterPro when they are considered to be of good quality; if two signatures are found to be describing the same protein domain, site or family, they are placed into the same database entry. Integrations are subject to rigorous checking and careful manual annotation in the form of both a written abstract and cross-references to the Gene Ontology. New in InterProScan 5 is the ability to output which pathways are associated with a particular InterPro entry (and, by extension, the protein under study). The link between pathway and entry is determined automatically, by checking if a significant proportion of proteins matched by an entry (>80%) are annotated with pathway-related information in UniProtKB. Enzyme Commission classifications [EC numbers, http://enzyme.expasy.org/; (Bairoch, 2000)] and cross-references to pathway resources such as KEGG [http://www.genome.jp/kegg/; (Kanehisa, 2013)] are currently available in InterProScan 5.

### 2.4 Match lookup service

As part of the regular release procedure used to generate the InterPro database at EMBL-EBI, matches are calculated for all UniParc protein sequences. These precalculated matches are made available to InterProScan via a lookup web service, to prevent unnecessary and wasteful recalculation. When a sequence is submitted to InterProScan for searching, a checksum of the amino acid sequence is calculated and this is used to query the lookup web service to see if that sequence has already been characterized. If it has been characterized, the results are returned to the user; if not, the required analysis jobs are launched. The lookup service comprises a large Oracle Berkeley DB (Java edition; http://www.oracle.com/technetwork/products/berkeleydb/overvieo/index-085366.html) database with a lightweight web application front-end, hosted at EMBL-EBI; it is able to handle multiple concurrent requests. There is also the option to download this web application and database to run locally, if required. This lookup service replaces the indexed match_complete.xml file used by InterProScan 4.

### 2.5 Installation and configuration

Previously, installation of InterProScan (4.*x*) was complex, involving the download of multiple files and running of an installation script, which frequently frustrated users. With InterProScan 5, we have simplified the installation by providing a complete distribution in a single compressed file, which, once uncompressed on a supported operating system, can be used immediately. If a user wishes to further configure the system to suit their local environment, they only need to edit a single configuration file, simplifying the setup process for the software.

## 3 INTERFACES AND ACCESS

**Fig. 4.**
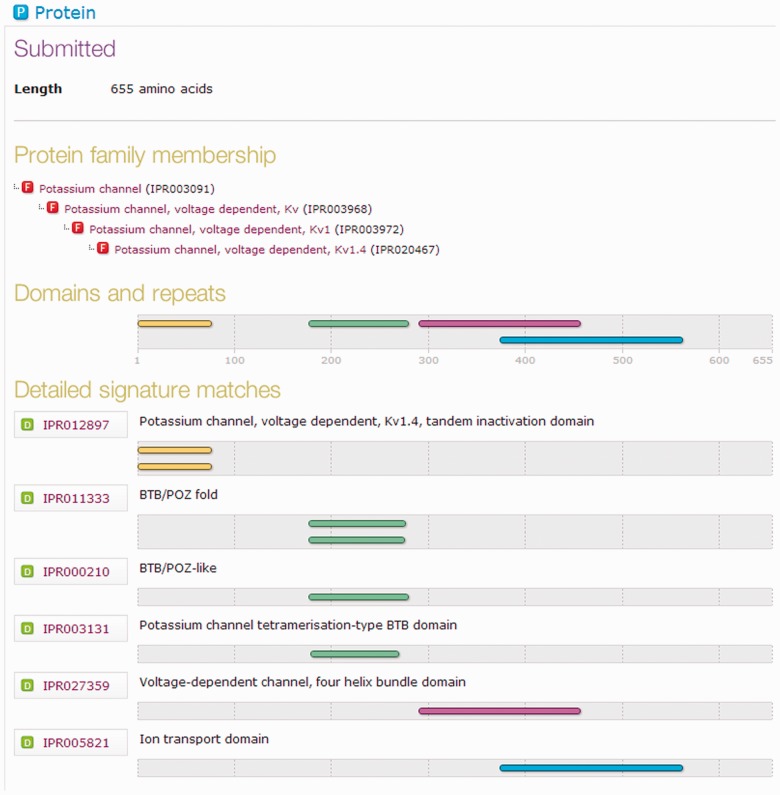
Portion of the graphical output from InterProScan 5. This view of a protein’s match data is the same in both the HTML and SVG formats

InterProScan 5 is primarily designed to run on the command-line. Previous downloadable versions included a web interface, but after a survey, we discovered this feature was only used by a small proportion of users. As the overhead for maintaining this interface was relatively high, it has been removed from version 5. However, users may still obtain a graphical view of their InterProScan 5 results by using the output format options -f HTML or -f SVG. This graphical view is now indistinguishable from the InterPro database’s protein pages (which display precomputed InterPro results against UniProtKB proteins), as shown in Figure 4.

Multiple other output formats are available, including tab-delimited (TSV), a new XML format and GFF3 (http://www.sequenceontology.org/resources/gff3.html). In particular, the new GFF3 format is useful for users wishing to map protein sequence feature predictions to both protein and nucleotide sequences (if nucleotide sequences were originally submitted to the search). Users wishing to output their results in a format compatible with version 4 of InterProScan (e.g. RAW) are able to use the ‘-convert’ mode on the InterProScan 5 XML output.

The InterProScan 5 distribution can be downloaded for local installation from the EMBL-EBI FTP site (ftp://ftp.ebi.ac.uk/pub/software/unix/iprscan/5/). Users can alternatively search their sequences using a hosted version via the InterPro Web site (http://www.ebi.ac.uk/interpro/) or SOAP (http://www.ebi.ac.uk/Tools/services/soap/iprscan5?wsdl) and REST (http://www.ebi.ac.uk/Tools/services/rest/iprscan5) web services (Goujon *et al.*, 2010). The source code is available from a Google Code repository (http://code.google.com/p/interproscan/), together with documentation for both developers and end-users.

It is possible to search both nucleotide and protein sequences with InterProScan (although the hosted version currently restricts users to being able to search a single protein (not nucleotide) sequence only, for band-width reasons).

## 4 CONCLUSIONS

InterProScan is a widely used tool for protein (and nucleotide) sequence analysis. InterProScan does not simply supply a convenient way of searching disparate analytical signatures from different source databases, but it also allows users to obtain a better overview of what their results mean by adding valuable information from the InterPro database.

This new implementation of InterProScan aims to address all of the shortcomings of the previous versions of InterProScan without losing important functionality. Primarily, the full reimplementation of the software into a robust, data model-driven, Java-based architecture gives considerable improvements in scalability and usability, with the new version much easier to install and configure. New features have been added, including the provision of an additional analysis algorithm (Phobius); new (GFF3, SVG) and improved (XML, HTML) output formats; new web services for lookup of precomputed results and the ability to infer the potential membership of proteins in pathways (via InterPro entry mappings to KEGG, MetaCyc, UniPathway, etc.). The hosting of the source code at Google Code also signifies a significant step toward InterProScan becoming a fully open source software development project. Future developments to the InterProScan software will be driven by the demands of the InterPro database and its users.
